# Investigation of Mucoadhesion and Degradation of PCL and PLGA Microcontainers for Oral Drug Delivery

**DOI:** 10.3390/polym11111828

**Published:** 2019-11-07

**Authors:** Zarmeena Abid, Mette Dalskov Mosgaard, Giorgio Manfroni, Ritika Singh Petersen, Line Hagner Nielsen, Anette Müllertz, Anja Boisen, Stephan Sylvest Keller

**Affiliations:** 1 The Danish National Research Foundation and Villum Foundation’s Center for Intelligent Drug Delivery and Sensing Using Microcontainers and Nanomechanics (IDUN), Technical University of Denmark, 2800 Kgs. Lyngby, Denmark; medmo@dtu.dk (M.D.M.); giomanfro94@gmail.com (G.M.); Risi@dtu.dk (R.S.P.); lihan@dtu.dk (L.H.N.); anette.mullertz@sund.ku.dk (A.M.); aboi@dtu.dk (A.B.); Suke@dtu.dk (S.S.K.); 2 National Centre for Nano Fabrication and Characterization, DTU Nanolab, Technical University of Denmark, 2800 Kgs. Lyngby, Denmark; 3 Department of Health Technology, DTU Health Tech, Technical University of Denmark, 2800 Kgs. Lyngby, Denmark; 4 Department of Pharmacy, Faculty of Health and Medical Sciences, University of Copenhagen, 2100 Copenhagen, Denmark

**Keywords:** hot punching, embossing, drug delivery, biodegradable polymers, thin films, microdevices, mucoadhesion, biodegradation

## Abstract

Microfabricated devices have been introduced as a promising approach to overcome some of the challenges related to oral administration of drugs and, thereby, improve their oral bioavailability. In this study, we fabricate biodegradable microcontainers with different polymers, namely poly-ɛ-caprolactone (PCL), poly(lactic-co-glycolic acid) (PLGA) 50:50 and PLGA 75:25 by hot punching. The mucoadhesion of the microcontainers is assessed with an *ex vivo* retention model on porcine intestinal tissue. Finally, *in vitro* degradation studies of the biodegradable microcontainers are completed for six weeks in simulated intestinal medium with the addition of pancreatic enzymes. Through SEM inspection, the PLGA 50:50 microcontainers show the first signs of degradation already after two weeks and complete degradation within four weeks, while the other polymers slowly degrade in the medium over several weeks.

## 1. Introduction

Oral drug delivery is the most preferred administration route due to its minimally invasive nature and high patient compliance. Moreover, it provides flexibility to accommodate various types of drug candidates, as oral dosage forms do not require sterile manufacturing conditions and are therefore less expensive to manufacture. However, oral drug delivery faces several challenges, such as pre-systemic intestinal degradation, hepatic first pass elimination, as well as low solubility of some drugs in the fluids of the gastrointestinal (GI) tract, leading to reduced oral bioavailability of the drug [1,2]. One goal in the development of oral drug delivery systems is to reduce the dosage and thereby minimize the adverse effects of the drug, which can be achieved by increasing the amount of drug specifically released and absorbed at the targeted site in the GI tract. In the last decades, mucoadhesion has been shown increased interest in oral drug delivery as it potentially enhances the bioavailability due to longer retention times of drug at the intended sites of absorption [3,4]. In particular, mucoadhesive drug delivery systems could be of value in delivering the growing number of sensitive high molecular-weight compounds, such as peptides and proteins [5]. Numerous methods have been proposed to assess the mucoadhesive properties of drug formulations *in vitro* and *ex vivo*. These methods can be based on mechanical force determination or on particle interactions [6].

Recently, microfabricated drug delivery systems have been proposed to overcome some of the major challenges in oral drug delivery [7]. For this purpose, microcontainer devices with precisely controlled dimensions and shapes have been introduced [8,9]. Microcontainers are reservoir-based devices, providing a large surface area. After the fabrication of microcontainers, active pharmaceutical ingredients (APIs) in various forms can be loaded into the cavities. This is convenient and avoids potential harm to the drug during the microcontainer processing steps. To control the release kinetics, functional coatings can then be applied on the loaded microcontainers. Due to the design of the devices, they can potentially provide unidirectional release at the intestinal mucosa, control of drug release kinetics, and facilitate targeted delivery of pharmaceuticals in the GI tract [10,11,12].

Based on the demand of fabricating microdevices in biocompatible and biodegradable materials, we recently demonstrated the successful fabrication of biodegradable microcontainers using hot punching, which is a modified hot embossing method [12]. The novel process is based on the assembly of compression molded polymer films, after which a single processing step produces simultaneous patterning of the device film and thermal bonding to an underlying water soluble poly(vinyl alcohol) (PVA) substrate. This results in replication of large arrays of microcontainers on a sacrificial film, which afterwards can be dissolved in an aqueous solution for harvesting of the devices. Thus far, this method has been applied for the fabrication of microcontainers with poly-ε-caprolactone (PCL). 

Recently, an *ex vivo* method was presented for characterization of the mucoadhesive properties of microcontainers and other microfabricated devices intended for oral drug delivery [13]. It is a simple, yet efficient method, where a capsule containing the devices is placed at the top end of an untreated and inclined piece of porcine small intestine. The tissue is perfused with a simulated intestinal medium followed by microscope examination. Using this method, a study was completed evaluating mucoadhesion of the PCL microcontainers fabricated by hot punching. The results showed good adhesion to the intestine with over 60% of the devices residing in the first few cm of intestine [13]. While this is promising for sustained drug delivery, it raises the concern that the microcontainers might accumulate in the GI tract upon repeated administrations. This illustrates that the degradation of the polymeric materials used for oral drug delivery applications is another critical factor to consider. PCL is a polyester with a relatively low degradation rate in physiological conditions [14,15]. By adjusting the fabrication process of biodegradable microcontainers, it could be possible to change the material to a biodegradable polymer with faster degradation and similar, or even better, mucoadhesive properties. A polymer that has attracted considerable interest as a base material in biomedical applications, due to its biocompatibility and tailorable biodegradation rate, is poly(lactic-co-glycolic acid) (PLGA) [16,17,18]. It is a co-polymer approved by the U.S. Food and Drug Administration (FDA) for several medical applications, such as drug delivery and tissue engineering, due to its non-toxicity in humans [16,18]. In the presence of an aqueous environment, PLGA undergoes hydrolytic degradation to produce lactic and glycolic acid, which are products of normal metabolic pathways in the human body. Based on the percentage of glycolic acid and lactic acid in PLGA, the physical properties, such as the hydration and hydrolysis rate, are different [18].

The aim of this work is to evaluate the suitability of different polymeric materials for microfabricated drug delivery devices where mucoadhesive properties and biodegradability are important. For this purpose, we fabricate microcontainers in PLGA 50:50, PLGA 75:25, and PCL with the hot punching process. Furthermore, we assess and compare the biodegradation *in vitro* and investigate the mucoadhesion of these microcontainers *ex vivo*. The degradation studies are completed in a simulated intestinal medium to evaluate the structural integrity of the microcontainers upon prolonged exposure. 

## 2. Materials and Methods 

### 2.1. Microcontainer Fabrication 

PVA (Mowiflex C17) was provided by Kuraray (Vantaa Finland) and PCL (M_n_ = 80,000 g mol^−1^) was purchased from Sigma Aldrich (Schnelldorf, Germany) . Circular polymer films with a diameter of approximately 100 mm were prepared by compression molding with a hot embosser (Collin® Press, 300 SV, Ebersberg, Germany) as described earlier [12]. 

A series of optimization steps were carried out in order to achieve uniform PLGA 50:50 (acid end cap, M_n_ 85,000–100,000 g/mol, Akina, IN, US) and PLGA 75:25 (acid end cap, M_n_ 75,000–85,000 g/mol, Akina, IN, US) films with thicknesses corresponding to the height of the desired microcontainer structures. For PLGA 50:50, compression molding temperature was varied between 70 and 90 °C and a film thickness of 83 ± 7 µm was achieved with the optimized parameters, as shown in Table 1. For PLGA 75:25, similar experiments were conducted where the temperature was varied between 90 and 110 °C, while the pressure and holding time were kept the same as for PLGA 50:50. The process was stopped at room temperature and a thickness of 86 ± 10 µm was achieved at a temperature of 105 °C.

**Figure 1:**
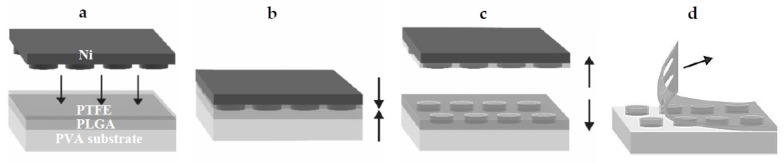
Schematic illustration of: (**a**) the assembly of poly(vinyl alcohol) (PVA) substrate film, a biodegradable PLGA device film, and a polytetrafluoroethylene (PTFE) film prior to the fabrication process; (**b**) hot punching is performed by applying pressure and heat; (**c**) a demolding step of the Ni stamp is completed, leading to separation of PLGA microcontainers from the surrounding film; (**d**) mechanical peeling of the surrounding PLGA film.

Nickel stamps with the microcontainer patterns were fabricated using dry etching and electroplating in a similar manner as described by Petersen et al. [19]. PLGA 50:50 and PLGA 75:25 microcontainers were fabricated using a similar process flow as for PCL microcontainers presented earlier [12]. A simple assembly of the compression molded polymer films was performed prior to a single step of simultaneous thermal bonding and patterning based on hot punching, as shown in Figure 1. A 28 × 28 mm^2^ PVA substrate was used as the sacrificial substrate and the PLGA device film with the same dimensions was assembled on top of it. Due to the high adhesion forces between PLGA and the Ni stamp, a polytetrafluoroethylene (PTFE) film (thickness 0.01 mm, Sigma Aldrich, Schnelldorf, Germany) was added. It was also cut in 28 × 28 mm^2^ to fit 4 × 400 microcontainers. The PLGA device film was molded and punched by the Ni stamp (80°C, 600 s, and platen pressure at 12 bars). After the punching process was finished, the temperature was decreased to 50°C with a cooling ramp of 20 °C min^−1^. Then, the stamp and the un-punched PTFE film were demolded from the polymer by mechanical peeling of the surrounding film, as shown in Figure 1D.

Optical profiler measurements were performed with a PLu neox 3D optical profilometer (Sensofar, Barcelona, Spain). Vertical scanning interferometry (VSI) measurements (20×) were conducted on five locations on the samples by attaching it to a silicon carrier wafer using Kapton tape in order to ensure a relatively planar surface prior to optical profiler measurements. Stylus profiler (Dektak XTA, Bruker, Karlsruhe, Germany) measurements were performed to ensure correct height determination by VSI. The data was analyzed using the free SPM data analysis software Gwyddion (version 2.52), and the data was levelled with respect to the indentations. Heights were determined based on profiles extracted across the center of the microcontainers. VSI scans were performed near the center and in each of the four corners of the samples. More detailed scans were also made for use in 3D rendering.

### 2.2. Harvesting of Biodegradable Microcontainers 

For harvesting of the PCL and PLGA microcontainers, the PVA substrate was dissolved. The dissolution of the sacrificial PVA was achieved by immersion in aqueous medium [12]. A sample containing 400 microcontainers was dissolved within 30 min. Subsequently, the free-floating microcontainers were harvested using a stainless steel filter with a mesh opening of 213 µm and thickness of 178 µm (Spectra/Mesh® Woven Filters, Fisher Scientific, Roskilde, Denmark). The microcontainers were dried at 37 °C for 1 h. For further investigation, the filter containing the harvested microcontainers was mounted on aluminum stubs with double-sided carbon adhesive dots. Scanning electron microscopy (SEM) was performed to study the surface and morphology of the microcontainers. All SEM micrographs were acquired by a TM3030Plus Tabletop Microscope (Hitachi, Krefeld, Germany) with a voltage of 15 keV using the SE detector.

### 2.3. Ex Vivo Mucoadhesion Studies with Biodegradable Microcontainers 

The *ex vivo* mucoadhesion studies were conducted as described by Mosgaard et al. [13] and conducted under the license number DK-10-13-oth-736416. An 18 cm piece of small intact intestinal porcine tissue was placed on an angled tissue holder at 20° inside a humidity and heat controlled chamber. The intestine was flushed with fasted state simulated intestinal fluid (FaSSIF, pH 6.5, 37 °C, Biorelevant®, London, UK) for 15 min with a flow rate of 4.1 mL/min using a peristaltic pump. Then, the angle was set to 10° and the tissue was flushed for 5 min at a flow rate of 1.55 mL/min. Gelatin capsules loaded with a known amount of microcontainers were placed at the top of the intestine and were allowed to dissolve for 15 min. The tissue holder was placed back at an angle of 20° and the intestine was perfused for 30 min at a flow rate of 1.55 mL/min. This flow rate was calculated based on earlier studies by Sinko et. al, reporting flow rates for rats of 0.2 mL/min and assuming a size ratio of 7.5 between a pig and rat intestine [20,21,22]. At the end of the study, the tissue was cut open and divided into 3 sections named “start”, “middle”, and “end”; each having a length of approximately 6 cm. The microcontainers found on the filter paper were referred to as “exit”. The tissue was transferred onto microscope slides and dried overnight at room temperature before visualization under a light microscope. The amount of microcontainers on each intestinal piece and the amount of microcontainers exiting the tissue were assessed.

### 2.4. Degradation Study in Intestinal Medium 

Degradation studies were performed in FaSSIF medium with added pancreatin enzymes, as they play a vital role in protein digestion in the small intestine and are the main enzymes used when simulating digestion [23]. Pancreatin from porcine pancreas (≥3 × USP specifications, Sigma Aldrich, St. Louis, MO, USA, 11.92 USPU/mg activity) was added to yield a lipase activity of 600 USPU/mL. The pancreatic extract was prepared by adding pancreatin to FaSSIF and vortexing until homogeneity was achieved. Afterwards, the mixture was centrifuged for 7 min at 4000 rpm and the supernatant was collected. The pH was adjusted to 6.5. The microcontainers were placed in 3 mL FaSSIF–pancreatin medium and the vials were kept in a 37°C waterbath with constant stirring (100 rpm). The medium was changed three times a week by filtering the microcontainers from the old medium. Once per week, the microcontainers were dried and investigated by SEM. The investigated samples were placed back in the vial before adding freshly prepared medium, and this was continued until no microcontainers could be found in the medium.

## 3. Results and Discussion 

### 3.1. Fabrication of PLGA Microcontainers 

**Figure 2:**
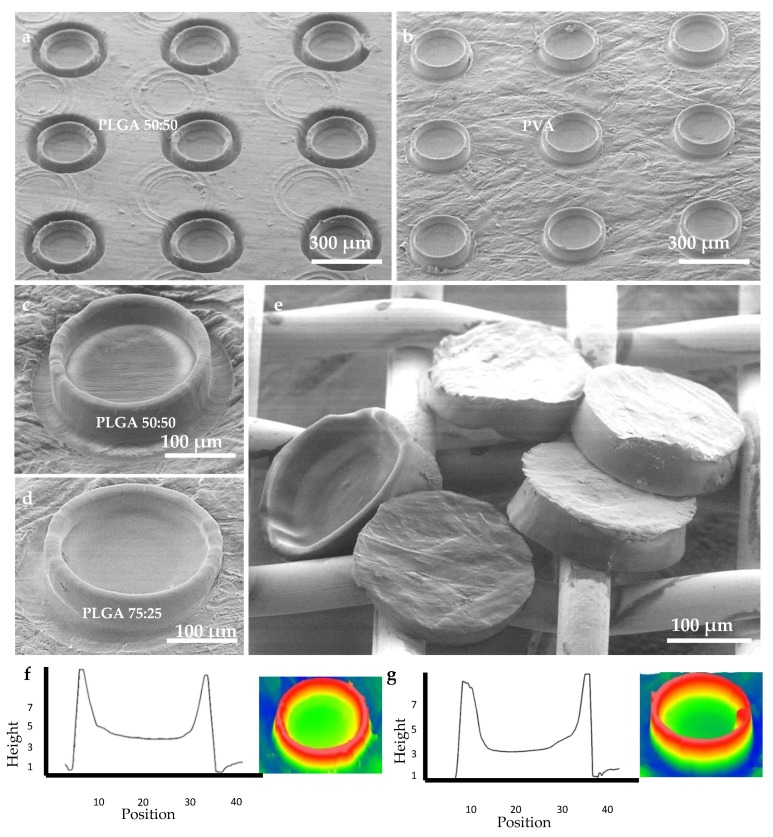
(**a**) SEM micrograph of microcontainer arrays before removal of the surrounding film; (**b**) microcontainer arrays after removal of the surrounding film; (**c**) close-up of a single PLGA 50:50 microcontainer; (**d**) close-up of a single PLGA 75:25 microcontainer; (**e**) harvested PLGA 50:50 microcontainers; (**f**) optical profile curve and 3D rendering of a single PLGA 50:50 microcontainer; (**g**) optical profile curve and 3D rendering of a single PLGA 75:25 microcontainer.

First, the fabrication of PLGA microcontainers by hot punching had to be optimized. The PLGA film was molded and finally punched due to shear stress at the highest protrusion of the stamp, which exceeded the ultimate tensile strength of the material, as shown in Figure 2a. By the addition of the PTFE film between the stamp and the PLGA device film, it was possible to avoid adhesion between the stamp and the PLGA device film upon demolding. After the hot punching process, the sacrificial PTFE film could easily be peeled off. The microcontainers were physically separated from the surrounding PLGA and remained on the underlying PVA substrate, as shown in Figure 2b–d. Microcontainers were successfully fabricated with PLGA 50:50 and 75:25 in arrays of 20 × 20 devices in a single-step hot punching process. The PVA substrate was dissolved in aqueous medium and the microcontainers were harvested on a grid, showing good structural definition and integrity, as shown in Figure 2e. SEM images revealed an excellent replication fidelity. The inner and outer diameter were 240 ± 2 µm and 275 ± 0.5 µm, respectively, the height was 73 ± 6 µm, and the reservoir depth was 56 ± 1 µm as investigated through optical profilometry and shown in Figure 2f,g. Compared to the PCL microcontainers fabricated with the identical Ni stamp [12], it was observed that the PLGA microcontainers were slightly lower. This was attributed to the PTFE film added during the hot punching step, which was not required for PCL. The inner diameter was 10 µm larger for PLGA microcontainers compared to PCL, which was expected as the PFTE had enlarged the stamp features. This led to different volumes of the PLGA and PCL microcontainers of 1.8 and 3.8 nL, respectively. The weight of a single microcontainer was determined experimentally by weighing a defined amount of devices. For PCL, the weight was 3.7 ± 0.4 µg, PLGA 50:50 had a weight of 2.7 ± 0.4 µg, and PLGA 75:25 weighed 3.6 ± 0.04 µg. This was in good agreement with the values estimated based on the measured dimensions and the polymer density.

### 3.2. Ex Vivo Mucoadhesion Study

**Figure 3:**
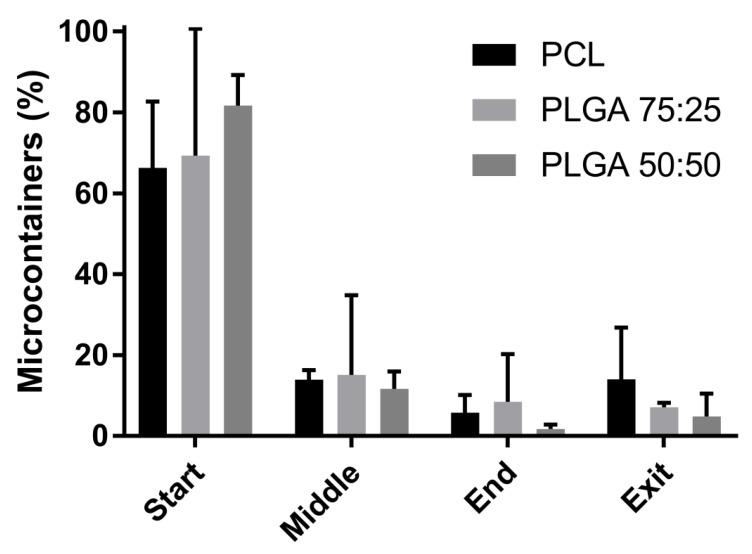
Percentage of microcontainers located in the start, middle, end, and exit of the small intestine of a pig after an *ex vivo* perfusion study. Comparison of poly-ɛ-caprolactone (PCL) (black) microcontainers, PLGA 75:25 (dark grey), and PLGA 50:50 (light grey). Data is presented as mean ± SD with n = 3–4.

After harvesting the microcontainers from the PVA substrate, *ex vivo* mucoadhesion tests on porcine small intestinal tissue were performed. The *ex vivo* retention test was used to evaluate the behavior of the microcontainers in the small intestine when exposed to a constant flow. The observation of the movement of the microcontainers down the small intestine, as shown in Supplementary Materials S2, indicates their interaction with the mucus layer. Prolonged movement of the microcontainers down the small intestine can be related to lower mucoadhesion. The recovery rate, which is the percentage of microcontainers that could be identified during the experiments, was 82 ± 6% for PLGA 50:50, 69 ± 10% for PLGA 75:25, and 88 ± 4% for PCL. It is assumed that the missing microcontainers were lost in a distributed manner through all intestinal sections and at the exit. A comparison of the relative percentages of PLGA 50:50, PLGA 75:25, and PCL microcontainers that could be identified in the respective sections is shown in Figure 3. It was observed that most of the microcontainers (between 66 and 82%) were located in the first part of the intestinal tissue, which indicates their ability to adhere well to the mucosal surface. The microcontainers were then more or less equally distributed throughout the rest of the intestinal sections. A slight tendency of PLGA microcontainers adhering better in the beginning of the tissue was observed compared to PCL microcontainers. A variety of factors could affect mucoadhesion, including the chemical structure which would lead to different interactions on the mucosal surface [24]. PLGA has a more hydrophilic structure than PCL, which can result in numerous hydrogen bonds with the mucus layer. PCL could, on the other hand, present hydrophobic interactions with mucus which explains the good ability to adhere in the beginning of the intestine [20,24]. Also, the size of the microcontainers could have an influence on the mucoadhesion. As the PLGA microcontainers had a slightly smaller diameter, they might have been less affected by the constant flow after adhering to the mucosal surface.

Minor variations between the two PLGA polymers were also observed. PLGA 50:50 microcontainers seemed slightly more prone to adhere to the mucus compared to PLGA 75:25. PLGA 50:50 has more hydrophilic functional groups, such as hydroxyl and carboxyl, which could again allow for better hydrogen bonding, thereby promoting maximal exposure of potential anchor sites. 

### 3.3. In Vitro Degradation Study in Intestinal Medium 

**Figure 4:**
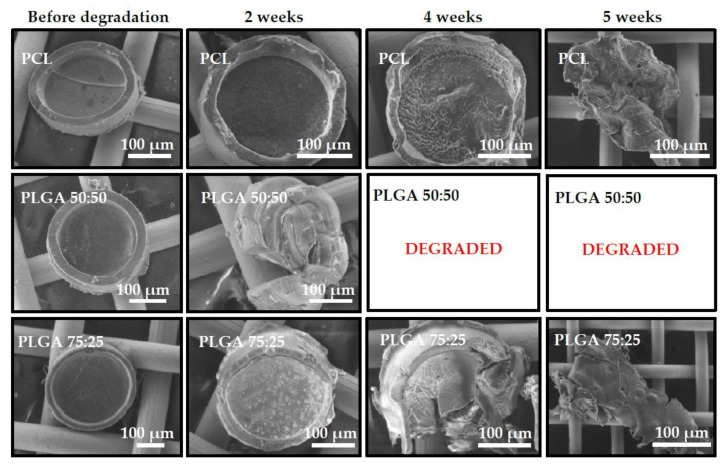
SEM micrographs of the morphology of microcontainers before degradation, after two weeks, four weeks, and six weeks, in simulated intestinal media, containing pancreatic enzymes.

The morphology of the microcontainers was analyzed by SEM before and during degradation in FaSSIF with pancreatin, as seen in Figure 4. PLGA 50:50 microcontainers already showed signs of degradation and loss of structural integrity in the first SEM analysis after two weeks. In comparison, PLGA 75:25 and PCL microcontainers only had minor deformation of the microcontainer walls in both cases. After four weeks, PLGA 50:50 microcontainers were completely degraded and thus, could not be detected anymore via SEM inspection. At the same time, PLGA 75:25 and PCL devices showed clear signs of degradation by changing structural integrity and even breaking apart. After five weeks, the morphology of those microcontainers had completely changed and only small polymer lumps remained. Finally, after six weeks, none of the three polymers could be detected through SEM. The observed degradation for all three polymers was faster compared to what would be expected from typical degradation studies reported in the literature. For PCL in an aqueous environment without any enzymes, degradation is typically slow, which would not allow complete degradation of the microcontainers within six weeks [14,15]. This is also supported by the fact that PCL microcontainers immersed in FaSSIF medium without enzymes were completely unaffected after 20 d, as shown in Supplementary Materials S1. The porcine pancreatin added in the FaSSIF medium contains a mixture of enzymes, including lipase, which readily hydrolyzes ester bonds in polyesters [23]. Furthermore, PCL is generally known to be degraded by microorganisms, as well as by hydrolytic mechanisms, under physiological conditions [25]. PLGA has been proposed to degrade primarily through hydrolytic degradation, but it has also been suggested that enzymatic degradation may play a role in the process [26,27]. Among all the tested polymers, PCL seemed to have the slowest biodegradation rate and literature also evidences that it is a slower degrading polymer in comparison to PLGA [26]. Regarding the difference between PLGA 50:50 and 75:25, it was expected that the degradation time would vary as different ratios of the monomers have a significant influence on hydrolysis and enzymatic degradation. PLGA 50:50 is expected to have the highest degradation rate compared to PLGA 75:25, which typically is approximately twice as slow due to the higher content of hydrophobic groups [27]. This corresponds well to what was observed during the degradation study. It should be noted, that *in vivo* degradation would be expected to occur even faster due to presence of bacteria and other enzymes in the GI tract.

## 4. Conclusions

In this study, mucoadhesion and degradation of polymeric microcontainers for oral drug delivery were investigated. For this purpose, the hot punching process for the fabrication of PLGA microcontainers had to be optimized. Due to high adhesion forces of PLGA polymer to the Ni stamp, an additional PTFE film was added between the stamp and the PLGA device film. This eased the demolding process and thus PLGA microcontainers in two different compositions, namely PLGA 50:50 and 75:25, were successfully fabricated. This demonstrates the versatility of the recently developed single-step hot punching method. The fabricated microcontainers were assessed in an *ex vivo* retention model for their mucoadhesion properties. It was found that PLGA 50:50 microcontainers showed the best mucoadhesion characteristics compared to PCL and PLGA 75:25 microcontainers. The degradation properties of the three types of biodegradable microcontainers were also evaluated in an *in vitro* study for six weeks using simulated intestinal medium with the addition of enzymes. Through SEM inspection, it was found that PLGA 50:50 degraded the fastest and no microcontainers could be detected already after four weeks. PCL and PLGA 75:25 microcontainers were completely degraded after six weeks. The results indicate that the fabrication method can indeed be used for various purposes of oral drug delivery and that PLGA 50:50 has the best mucoadhesion and the fastest biodegradation.

## Figures and Tables

**Table 1:** Parameters for compression molding of poly(lactic-co-glycolic acid) (PLGA) 50:50 and 75:25 films.

Material	Amount [mg]	Compression Time [min]	Holding Temperature [°C]	Cooling Ramp [K/min]	Platen Pressure [bar]
PLGA 50:50	250 ± 20	30	90	20	20
PLGA 75:25	400 ± 200	30	105	20	20

## Supplementary Materials

The following are available online at https://www.mdpi.com/2073-4360/11/11/1828/s1, Figure S1: Degradation study of PCL microcontainers in PBS, PaSSGF and FaSSIG media for up to 20 days; Figure S2: Microcontainers inside the porcine intestine visualized with an optical microscope.
