# Supplementary material for: Investigation of Mucoadhesion and Degradation of PCL and PLGA Microcontainers for Oral Drug Delivery
Source: Polymers (Basel). 2019 Nov 7;11(11):1828. doi: 10.3390/polym11111828 (PMC6918296; doi:10.3390/polym11111828)
Supplement: Supplementary file 1 [file polymers-11-01828-s001.pdf]

polymers

MDPI

Supplementary Materials: Investigation of Mucoadhesion and Degradation of PCL and PLGA Microcontainers for Oral Drug Delivery

Zarmeena Abid [1,2,*], Mette Dalskov Mosgaard [1,3], Giorgio Manfroni [1,2], Ritika Singh Petersen [1,2], Line Hagner Nielsen [1,3], Anette Müllertz [1,4], Anja Boisen [1,3] and Stephan Sylvest Keller [1,2]

1 The Danish National Research Foundation and Villum Foundation's Center for Intelligent Drug Delivery and Sensing Using Microcontainers and Nanomechanics (IDUN), Technical University of Denmark, 2800 Kgs. Lyngby, Denmark; medmo@dtu.dk (M.D.M.); giomanfro94@gmail.com (G.M.); Risi@dtu.dk (R.S.P.); lihan@dtu.dk (L.H.N.); anette.mullertz@sund.ku.dk (A.M.); aboi@dtu.dk (A.B.); Suke@dtu.dk (S.S.K.)

2 National Centre for Nano Fabrication and Characterization, DTU Nanolab, Technical University of Denmark, 2800 Kgs. Lyngby, Denmark

3 Department of Health Technology, DTU Health Tech, Technical University of Denmark, 2800 Kgs. Lyngby, Denmark

4 Department of Pharmacy, Faculty of Health and Medical Sciences, University of Copenhagen, 2100 Copenhagen, Denmark

* Correspondence: zarab@dtu.dk

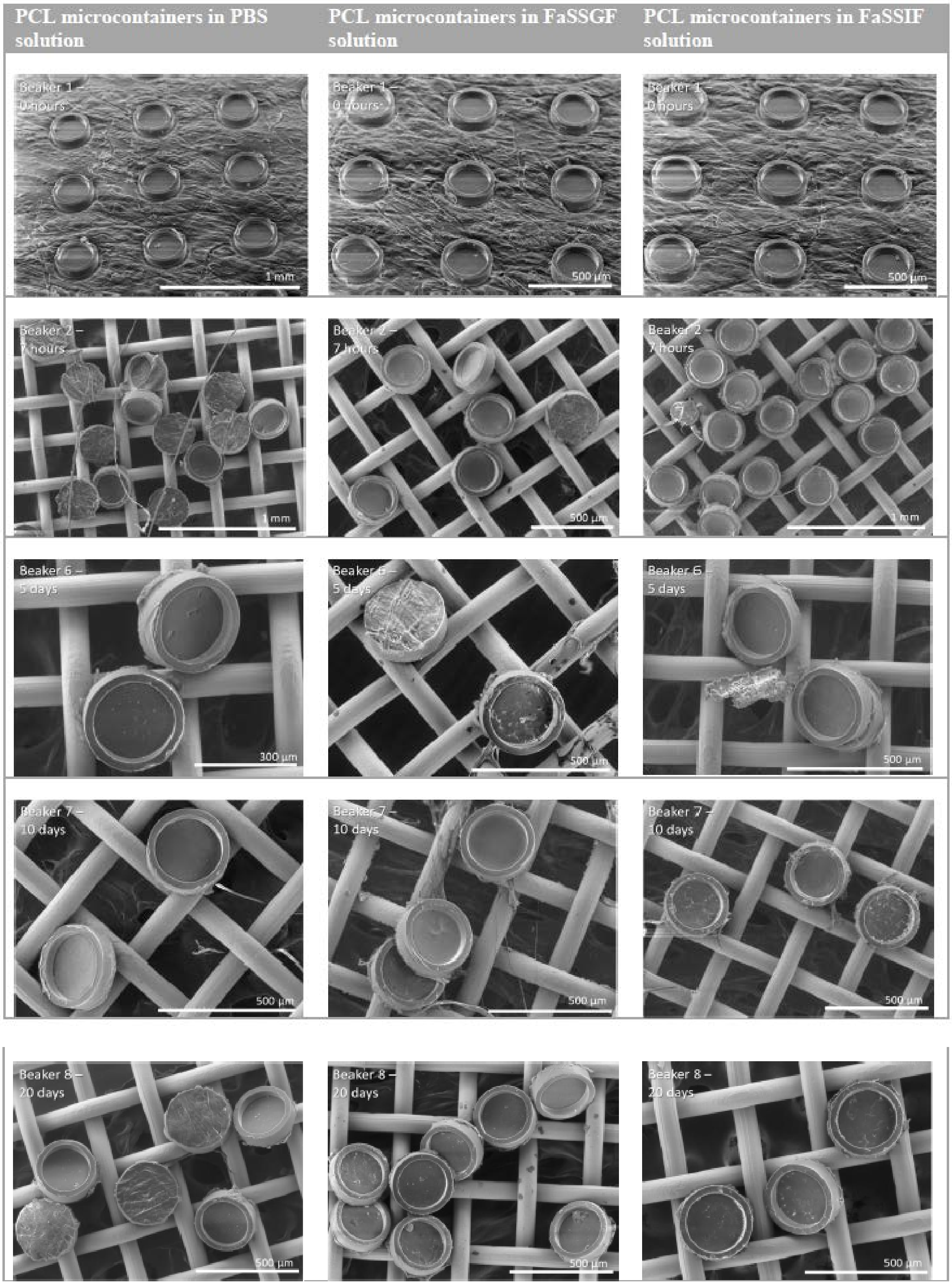

Figure 1. Degradation study of PCL microcontainers in PBS, FaSSGF and FaSSIF media for up to 20 days.

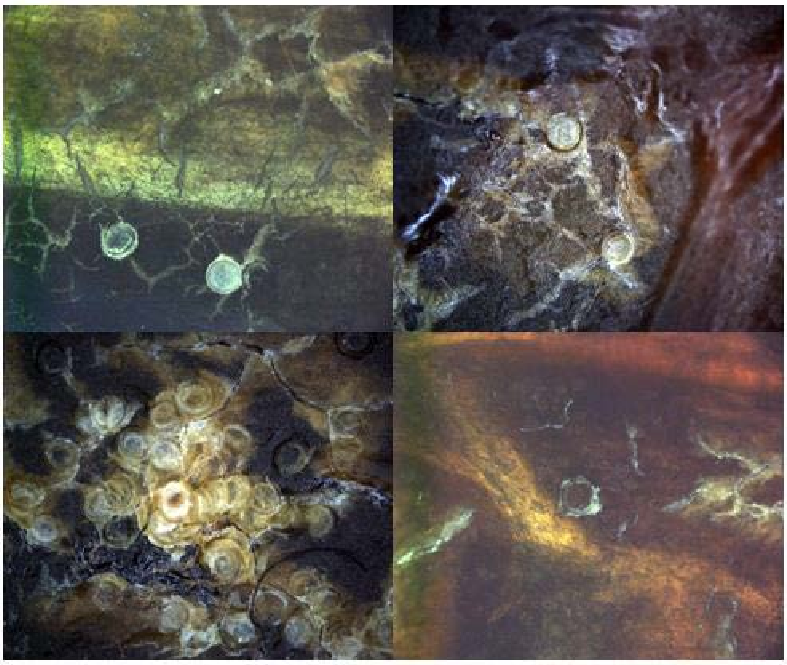

Figure S2. Microcontainers inside the porcine intestine visualized with an optical microscope.

© 2019 by the authors. Submitted for possible open access publication under the terms and conditions of the Creative Commons Attribution (CC BY) license (http://creativecommons.org/licenses/by/4.0/).
